# Loss of *Perp* in T Cells Promotes Resistance to Apoptosis of T Helper 17 Cells and Exacerbates the Development of Experimental Autoimmune Encephalomyelitis in Mice

**DOI:** 10.3389/fimmu.2018.00842

**Published:** 2018-04-23

**Authors:** Yan Zhou, Xiao Leng, Yan He, Yan Li, Yuan Liu, Yang Liu, Qiang Zou, Guixiu Shi, Yantang Wang

**Affiliations:** ^1^Department of Emergency, West China Second University Hospital and Key Laboratory of Obstetric and Gynecologic and Pediatric Diseases and Birth Defects, Ministry of Education, Sichuan University, Chengdu, China; ^2^Department of Immunology, School of Basic Medical Sciences, Chengdu Medical College, Chengdu, China; ^3^Department of Rheumatology and Clinical Immunology, The First Affiliated Hospital, Xiamen University, Xiamen, China

**Keywords:** *Perp*, T helper 17 cell, activation-induced cell death, experimental autoimmune encephalomyelitis/multiple sclerosis, caspase activation

## Abstract

T helper 17 (Th17) cells are crucial for the pathogenesis of multiple sclerosis (MS) in humans and experimental autoimmune encephalomyelitis (EAE) in animals. High frequency of Th17 cells and low sensitivity to activation-induced cell death (AICD) are detected in MS patients. However, the mechanisms underlying apoptosis resistance of T cells remain unclear. *Perp* is an apoptosis-associated target of *p53* and implicated in the development of cancers. Here, we show that loss of *Perp* in T cells does not affect Th1, Th17, or Treg cell differentiation, but does significantly increase the resistance of *Perp*^−/−^ Th17 cells to AICD and anti-Fas in Lck-Cre × *Perp*^fl/fl^ mice by inhibiting the caspase-dependent apoptotic pathway. Moreover, Lck-Cre × *Perp*^fl/fl^ mice exhibited earlier onset of EAE and severe spinal cord inflammation and demyelination, accompanied by increased levels of pro-inflammatory cytokines and enlarged population of Th17 cells. Therefore, *Perp* deletion promoted Th17 responses and exacerbated the development and severity of EAE.

## Introduction

T helper 17 (Th17) cells are crucial for the pathogenesis of autoimmune diseases (AID), such as multiple sclerosis (MS) and its experimental model, experimental autoimmune encephalomyelitis (EAE) (1, 2). Initially, IL-17A and IL-22 produced by Th17 cells can disrupt tight junctions at the blood–brain barrier and facilitate the penetration of autoreactive Th17 and Th1 cells into the central nervous system (CNS) (3). Subsequently, autoreactive Th1 and Th17 cells interact with myeloid cells and B cells, together with their secreting pro-inflammatory cytokines, to create a microenvironment, leading to a sustained myelin and axonal damage in the CNS (4). Actually, a high frequency of Th17 cells is detected in peripheral blood and the CNS lesions of patients with active MS (5, 6). In addition, adoptive transfer of myelin-specific Th17 cells induces EAE in naive syngeneic animals (7). Hence, the maintenance of Th17 cell homeostasis is important for the inhibition of autoimmune demyelination. It is well known that activated T cells can undergo apoptosis, or activation-induced cell death (AICD), following repeated T-cell receptor (TCR) stimulation and through the Fas/FasL signaling. Such self-regulation can eliminate excessive and prolonged presence of effector T cells and help in maintaining peripheral tolerance (8). Alternation in AICD of T cells is associated with the development of AID and low levels of AICD in T cells are observed in patients with relapsing-remitting MS (9). However, the precise mechanisms underlying the resistance of Th17 cells to AICD in MS patients have not been clarified.

PERP, an apoptosis-associated target of *p53*, is a membrane protein of the PMP-22/gas3 family and is implicated in the development of a variety of cancers (10). The *Perp^−^*^/^*^−^* mice die postnatally with extensive blistering on the skin and oral mucosa, the lethal phenotype that indicated an important role of PERP in the *p63*-directed epithelial development (11, 12). Furthermore, PERP also regulates the apoptosis of thymocytes and peripheral T cells through the *p53* apoptotic pathway. Activation of murine T cells by antigen stimulation upregulates the expression of PERP, contributing to their apoptosis (13). Modulation of PERP expression by a CD25 blockade decreases the FasL-mediated AICD of human T cells (14). Furthermore, CD4^+^CD8^+^ thymocytes from *Perp^−^*^/^*^−^* mice are resistant to radiation-induced apoptosis (15). In addition, decreased levels of PERP expression are detected on peripheral blood mononuclear cells (PBMCs) from patients with rheumatic arthritis (RA), and the levels of PERP expression are inversely correlated with IL-17 responses and disease activity (16). Accordingly, we hypothesize that *Perp^−^*^/^*^−^* in T cells may inhibit AICD of Th17 cells to exacerbate the development of EAE.

In this study, we generated the Lck-Cre × *Perp*^fl/fl^ mice *with Perp^−^*^/^*^−^* in T cells and examined the impact of *Perp^−^*^/^*^−^* in T cells on Th1, Th17, or Treg cell differentiation and apoptosis as well as potential apoptosis pathway *in vitro*. Furthermore, we investigated the effect of *Perp^−^*^/^*^−^* in T cells on the development of EAE in mice. Our data indicated that *Perp^−^*^/^*^−^* in T cells did not affect Th1, Th17, or Treg cell differentiation, but did increase the resistance to anti-Fas induced apoptosis in Th17 cells, accompanied by inhibiting the caspase-dependent pathway *in vitro*. *Perp^−^*^/^*^−^* in T cells promoted the early onset and severity of EAE by increased levels of inflammation and demyelination in the CNS, which was associated with enhanced Th17 responses *in vivo*. Our findings may provide new insights in the regulation of Th17 responses and pathogenesis of EAE.

## Materials and Methods

### Mice

*Perp*^fl/+^ mice were purchased from the Jackson Laboratory (Stock No: 016132, Bar Harbor, ME, USA). Lck-Cre mice were obtained from Model Animal Research Center of Nanjing University (Nanjing, China). The *Perp*^fl/fl^ and Lck-Cre mice were bred to obtain littermate control, Lck-Cre × *Perp*^fl/+^ and Lck-Cre × *Perp*^fl/fl^ mice. All mice were housed in a specific pathogen-free facility at the Experimental Animal Center of Chengdu Medical College. The experimental protocol was approved by the Animal Care and Use Committee of Chengdu Medical College. C57BL/6 mice with EAE were used as an animal model of human MS.

### Breeding and Identification of Lck-Cre × *Perp*^fl/+^ and Lck-Cre × *Perp*^fl/fl^ Mice

To obtain the mice with *Perp^−/−^* specific in T cells, male and female *Perp*^fl/+^ mice were mated with a ratio of 1:1. The F1 *Perp*^fl/fl^ mice were identified by PCR and bred with Lck-Cre mice to generate the Lck-Cre × *Perp*^fl/+^ mice. Subsequently, the male and female Lck-Cre × *Perp*^fl/+^ mice were bred to obtain the Lck-Cre × *Perp*^fl/fl^ mice. Their genotypes were analyzed by PCR and Western blot. The sequences of PCR primers were forward 5′-AGT CTT CAG GGA TGA CAC AGA-3′ and reverse, 5′-TAC GAA ACT AGA GCA CAG CTA-3′ for *Perp*; forward 5′-ATT TGC CTG CAT TAC CGG TC-3′ and reverse, 5′-ATT TGC CTG CAT TAC CGG TC-3′ for Lck-Cre.

### Murine T Cell Isolation and Differentiation

Naïve splenic CD4^+^ T cells were isolated from 8-week-old female littermate control, Lck-Cre × *Perp*^fl/+^ and Lck-Cre × *Perp*^fl/fl^ mice as described previously (17) and purified using the Naive CD4^+^ T Cell Isolation Kit (Miltenyi-Biotec, Bergisch Gladbach, Germany). The purified cells were characterized by FACS. The purified naive CD4^+^ T cells (1 × 10^6^ cells/ml) were stimulated with immobilized anti-CD3 (2 µg/ml) and soluble anti-CD28 (2 µg/ml, BD PharMingen, San Diego, CA, USA) in RPMI 1640 containing 10% FCS. To evaluate the effect of *Perp^−/−^* on the differentiation of Th17 cell, the naïve CD4^+^ T cells were stimulated with anti-CD3 and anti-CD28 in the presence of recombinant human TGF-β1 (5 ng/ml, PeproTech, Rocky Hill, NJ, USA), IL-6 (20 ng/ml, PeproTech), anti-IL-4 (10 µg/ml), and anti-IFN-γ (20 µg/ml BD PharMingen) for 3 days. For Th1 differentiation, naïve CD4^+^ T cells were stimulated with anti-CD3 and anti-CD28 in the presence of anti-IL-4 (10 µg/ml), and recombinant mouse IL-12 (10 ng/ml, PeproTech) for 3 days. For Treg differentiation, naïve CD4^+^ T cells were stimulated with anti-CD3 and anti-CD28 in the presence of anti-IL-4 (10 µg/ml), anti-IFN-γ (20 µg/ml), and recombinant human TGF-β1 (2 ng/ml) for 3 days. The cells were washed with PBS and used for subsequent experiments.

### Intracellular Staining and Flow Cytometry

The frequency of different subsets of Th cells was characterized by FACS. Briefly, the cells were stained with fluorescein isothiocyanate (FITC)-anti-CD4, fixed, and permeabilized with GolgiPlug™ (BD PharMingen). After being washed, the cells were stained intracellularly with PE-conjugated anti-IFN-γ and Alexa Fluor^®^ 647-conjugated anti-IL-17, followed by FACS analysis. Some splenocytes were stained with FITC-anti-CD4 and APC-anti-CD25 (BD PharMingen), fixed, permeabilized, and stained with PE-anti-Foxp3 (BD PharMingen), followed by FACS analysis of the frequency of Tregs. Some splenocytes were stained with FITC-anti-CD4 and PE-anti-CD44 (BD PharMingen), fixed, permeabilized, and stained with Alexa Fluor^®^ 647-conjugated anti-IL-17 (BD PharMingen), followed by FACS analysis of the frequency of memory Th17 cells.

### Apoptosis

The naïve T cells were stimulated with anti-CD3/anti-CD28 in the presence of Th1, Th17, or Treg polarizing cytokine-cocktail and neutralizing antibodies for 5 days. The cells were reactivated with anti-CD3 (2 µg/ml) for 72 h in the presence or absence of 1 µg/ml anti-Fas (BD PharMingen). The percentages of Annexin V^+^7-AAD^−^ apoptotic Th1, Th17, or Treg cells were analyzed by FACS using an Annexin V apoptosis detection kit (BD PharMingen), according to the manufacturer’s instructions.

### Western Blot

The differentiated Th17 cells from littermate control, Lck-Cre × *Perp*^fl/+^ and Lck-Cre × *Perp*^fl/fl^ mice were lysed in RIPA buffer (Millipore, Billerica, MA, USA) and centrifuged. The protein concentrations in the lysates were determined using a BCA Protein Assay Kit (Pierce, Rockford, IL, USA). The mitochondria and cytosol of some differentiated Th17 cells were prepared using the Mitochondria/Cytosol Fractionation kit (Abcam). The cell lysate (50 μg/lane) was separated by sodium dodecyl sulfate polyacrylamide gel electrophoresis (SDS-PAGE) on 12% gels and transferred onto the polyvinylidene difluoride membrane. The membranes were blocked with 5% non-fat dry milk in PBST and incubated with primary antibodies against PERP (Santa Cruz Biotechnology, Santa Cruz, CA, USA), cleaved caspase-8, Bcl-2, Bcl-xL, cytochrome *c* (cyto-*c*), cleaved caspase-3 (Cell Signaling Technology, Beverly, MA, USA), and tubulin (ZSBiO, Beijing, China) at 4°C overnight. After being washed, the bound antibodies were detected with horseradish peroxidase-conjugated goat anti-rabbit IgG (Cell Signaling Technology) and visualized by the ChemiDoc XRS (Bio-Rad, Hercules, CA, USA). The relative levels of target protein to the control tubulin were determined by densitometric analyses using Quantity One software (Bio-Rad).

### Induction and Evaluation of EAE

The littermate control, Lck-Cre × *Perp*^fl/+^ and Lck-Cre × *Perp*^fl/fl^ mice at 10 weeks of age were immunized with 200 µg MOG_35–55_ (Chinese Peptide, Shanghai, China) emulsified in 50% complete Freud’s adjuvant (CFA) containing 4 mg/ml heat-killed *Mycobacterium tuberculosis* (Chondrex, Redmond, WA, USA) at their dorsal flanks. Individual mice were injected intraperitoneally with pertussis toxin (200 ng/mouse, Millipore) on day 0 and 2. The development and severity of EAE in individual mice were scored daily using the following score system: 0, healthy; 1, tail paralyzed; 2, no coordinated movement; hind limb paresis; 3, both hind limbs paralyzed; 4, forelimbs paralyzed; and 5, moribund state.

### Histology

At 23 days post-induction, blood samples were collected from individual mice. The mice were anesthetized by 2% pentobarbital sodium and perfused intracardially with PBS (pH 7.4) followed by 4% paraformaldehyde in PBS. Their spinal cord samples were dissected. Some tissues from each group were fixed in 4% paraformaldehyde overnight and paraffin embedded. The sagittal cervicothoracic spinal cord sections (5 µm) were stained with H&E and Luxol fast blue to examine the degrees of inflammation and demyelination, respectively.

### RNA Extraction and Real-Time PCR

Total RNA was extracted from the CNS tissues in individual groups of mice using Trizol (Invitrogen), following the manufacturer’s instructions. Subsequently, 2 µg total RNA of each sample was reversely transcribed to cDNA using the iScript™ Advanced cDNA Synthesis Kit, according to the manufacturer’s protocol (Bio-Rad). The relative levels of TNF-α, IL-6, and IL-17A mRNA transcripts to GAPDH were determined by quantitative real-time PCR (CFX 96, Bio-Rad) using iQ™ SYBR^®^ Green Supermix (Bio-Rad) and specific primers. The sequences of primers were sense 5′-TGGCTAAGGACCAAGACCATCCAA-3′ and antisense 5′-AACGCACTAGGTTTGCCGAGTAGA-3′ for IL-6; sense 5′-GCTCCAGAAGGCCCTCAGA-3′ and antisense 5′-AGCTTTCCCTCCGCATTGA-3′ for IL-17A; sense 5′-CCTCCCTCTCATCAGTTCTATGG-3′ and antisense 5′-CGTGGGCTACAGGCTTGTC-3′ for TNF-α; and sense 5′-GGCAAATTCAACGGCACA-3′ and antisense 5′-GTTAGTGGGGTCTCGCTCTG-3′ for GAPDH. The levels of mRNA transcripts were normalized to GAPDH and analyzed by the 2^−ΔΔCt^ method.

### Immunofluorescent Staining

Some spinal cord tissues from each group were fixed in 4% paraformaldehyde overnight, immersed in 30% sucrose, and embedded in OCT on dry ice. The cryostat transversal lumbar spinal cord sections (20 µm) were washed twice with PBS and blocked with 10% normal mouse serum in PBS for 1 h at room temperature. The sections were incubated with goat anti-mouse IL-17A (Santa Cruz Biotechnology) or rat anti-mouse CD4 (BD PharMingen) overnight at 4°C. After being washed, the sections were incubated with PE-anti-goat IgG (Santa Cruz Biotechnology) and Alexa Fluor 488-anti-rat IgG (Abcam). Images were taken under an Olympus Confocal FV1000 microscope.

### ELISA

The concentrations of serum IL-1β, IL-6, IL-10, IL-23, TNFα, and IL-17A in individual mice were determined using the cytokine-specific ELISA kits (eBioscience, San Diego, CA, USA), according to the manufacturer’s instructions.

### Statistical Analysis

Data are expressed as the mean ± SEM. The difference between two groups was evaluated by two-tailed unpaired Student’s *t*-test (Gaussian distribution) or two-tailed Mann–Whitney *U*-test (no Gaussian distribution). The difference among three or more groups was analyzed by repeated ANOVA and *post hoc* Bonferroni correction. All statistical analyses were performed using PRISM 6.0 software (GraphPad Software, San Diego, CA, USA). A *P*-value of <0.05 was considered statistically significant.

## Results

### Propagation and Genotype Identification of the Transgenic Mice

**Figure 1:**
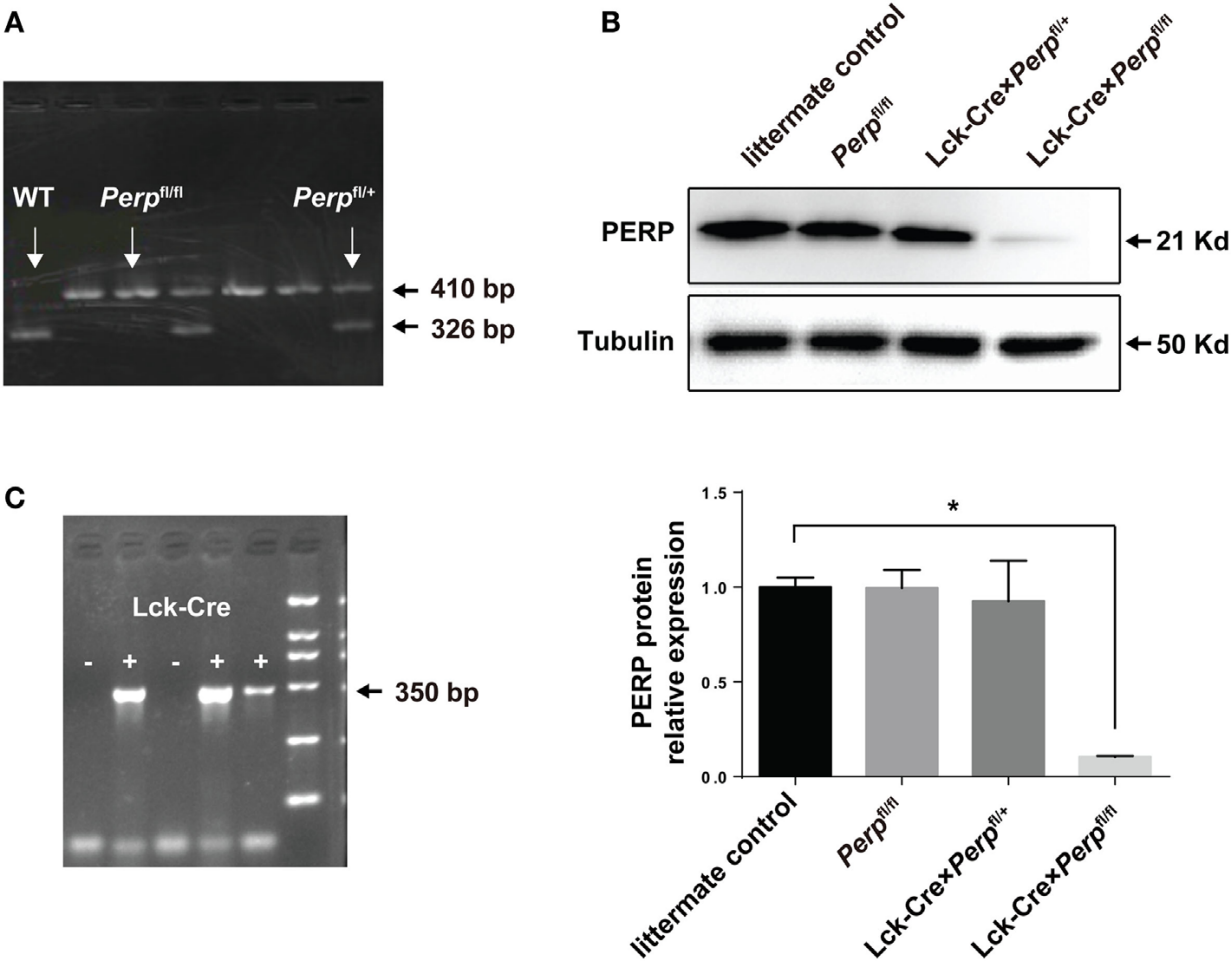
Identification of Lck-Cre × *Perp*^fl/+^ and Lck-Cre × *Perp*^fl/fl^ mice. Following breeding *Perp*^fl/fl^ mice, the F1 Lck-Cre × *Perp*^fl/+^ and Lck-Cre × *Perp*^fl/fl^ mice were identified by PCR analysis of the genomic DNA from peripheral blood mononuclear cell for the *Perp* **(A)** and Lck-Cre **(C)** genes and Western blot analysis of splenic T cells **(B)**. Data are representative images or expressed as the mean ± SEM of each group from three separate experiments (*P < 0.05).

To evaluate the impact of *Perp^−/−^* in T cells on the Th17-cell survival and pathogenesis of EAE, the Lck-Cre × *Perp*^fl/+^ and Lck-Cre × *Perp*^fl/fl^ mice were generated and their genotypes on CD4^+^ T cells were identified by PCR (Figures 1A,C). Western blot analysis indicated that there was almost no detectable PERP expression in CD4^+^ T cells from Lck-Cre × *Perp*^fl/fl^ mice (Figure 1B), but the levels of PERP expression in CD4^+^ T cells from Lck-Cre × *Perp*^fl/+^ mice were only slightly lower than that in the littermate control mice (Figure 1B). These two stains of mice were valuable for evaluating the importance of PERP in the survival of Th17 cells and the development of EAE.

### Deficiency of *Perp* Inhibits Th17 Cell Apoptosis, But Does Not Affect Their Differentiation

**Figure 2:**
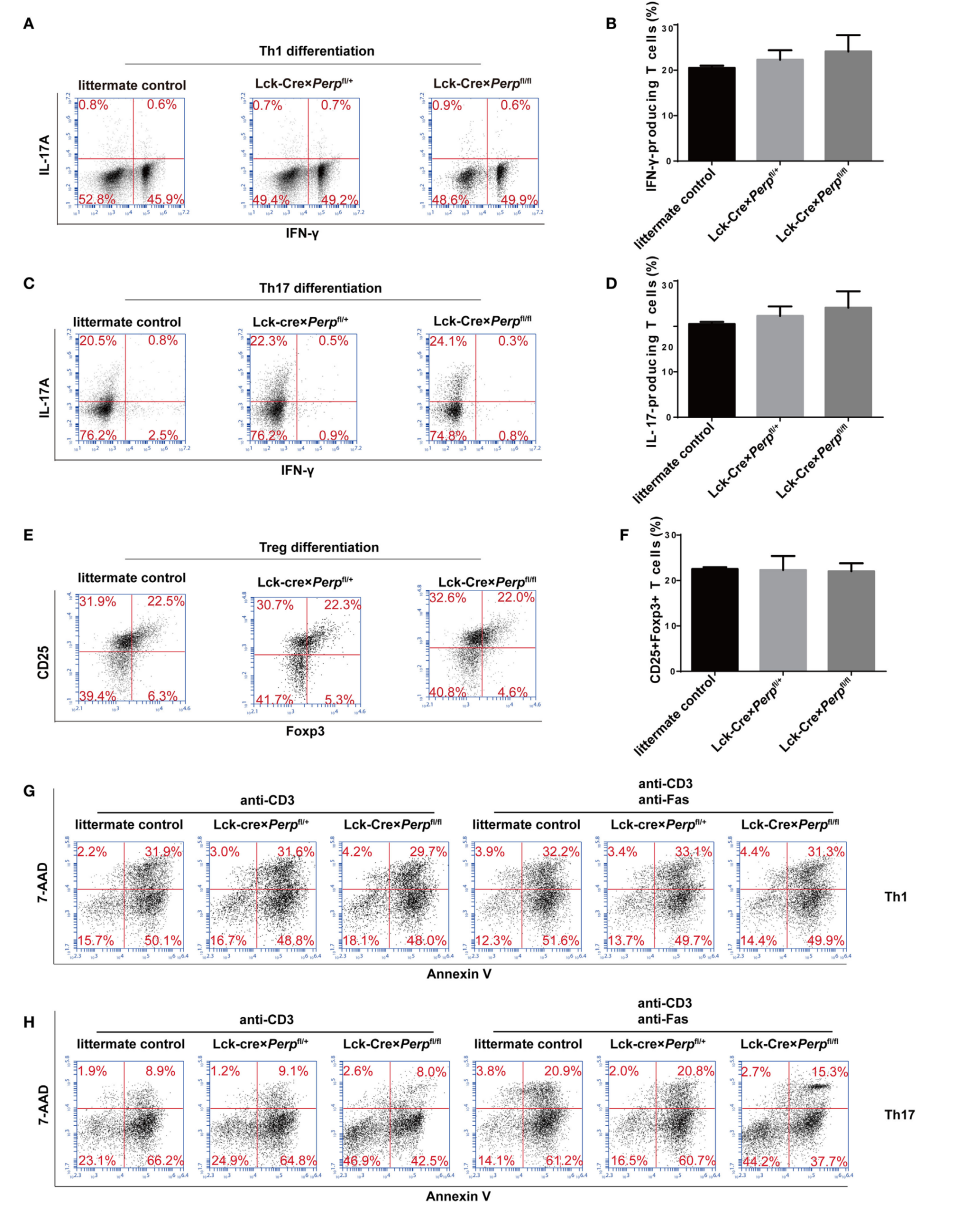

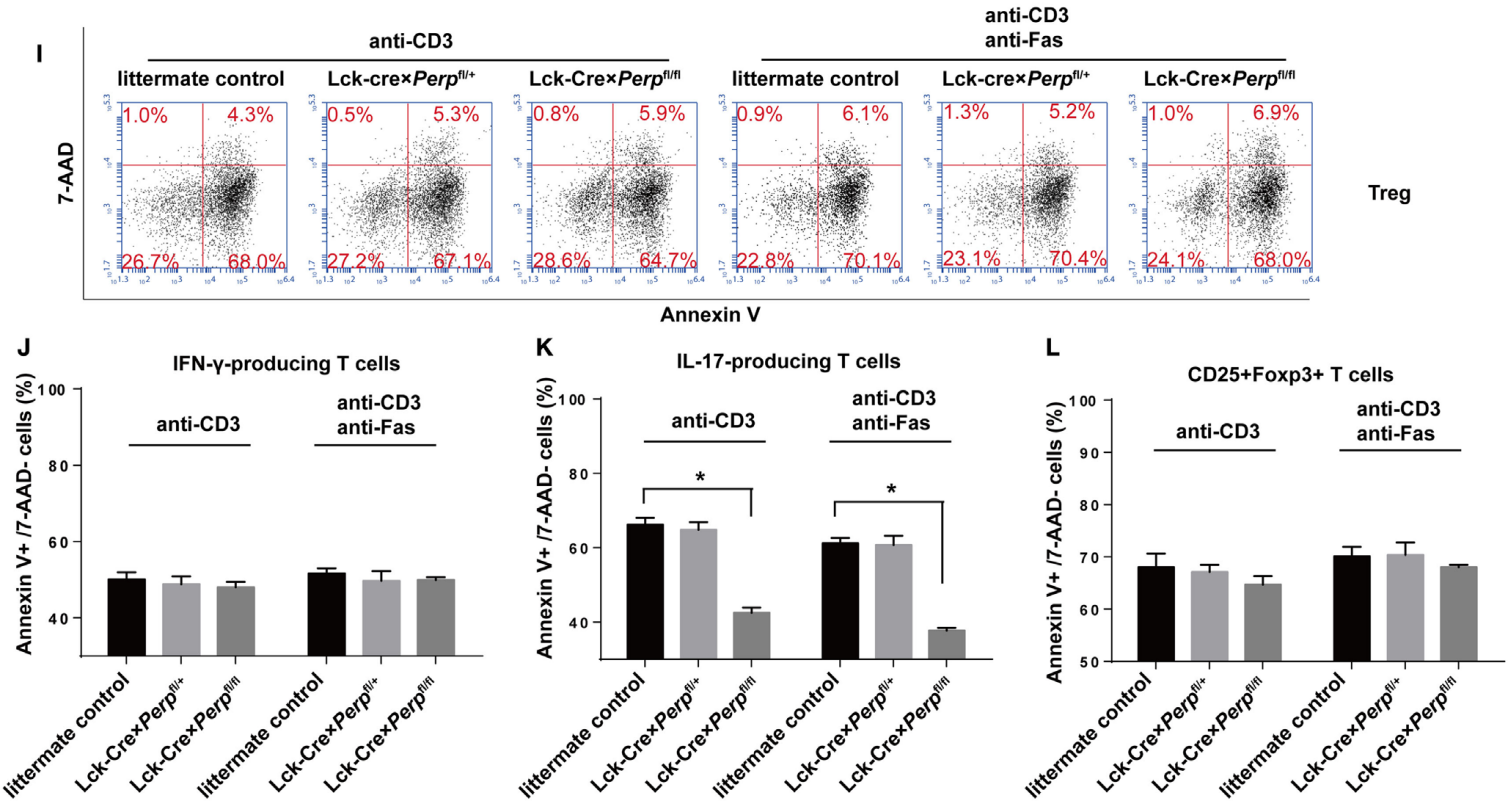
*Perp^−/−^* in T cells promotes the resistance to apoptosis in T helper 17 (Th17) cells. CD4^+^ T cells were isolated from littermate control, Lck-Cre × *Perp*^fl/+^ and Lck-Cre × *Perp*^fl/fl^ mice by negative selection and stimulated with anti-CD3/anti-CD28 in the presence of cocktail of cytokines and antibodies to induce Th1, Th17, or Treg cell differentiation for 3 days. The cells were stained with anti-CD4 and/or anti-CD25, fixed, permeabilized, and intracellularly stained with anti-IFN-γ, anti-IL-17A, or anti-Foxp3. The frequency of Th1, Th17, or Treg cells was determined by FACS. Furthermore, following *in vitro* differentiation for 3 days, the cells were re-stimulated with anti-CD3/anti-CD28 in the presence or absence of anti-Fas for 72 h. The percentages of apoptotic Th1, Th17, or Treg cells were determined by FACS using Annexin V-PE and 7-AAD staining. Data are representative images or expressed as the mean ± SEM of each group from three separate experiments. **(A,B)** Th1 cell differentiation. **(C,D)** Th17 cell differentiation. **(E,F)** Treg cell differentiation. **(G,J)** Th1 cell apoptosis. **(H,K)** Th17 cell apoptosis. **(I,L)** Th17 cell apoptosis (*P < 0.05).

To investigate the impact of *Perp^−/−^* on Th1, Th17, or Treg differentiation and survival, naïve CD4^+^ T cells from littermate control, Lck-Cre × *Perp*^fl/+^ and Lck-Cre × *Perp*^fl/fl^ mice were purified and stimulated with anti-CD3/anti-CD28 in the presence of cocktails of cytokines and antibodies to induce Th1, Th17, or Treg cell differentiation for 3 days. The percentages of Th1, Th17, or Treg cells were analyzed by FACS. There was no significant difference in the frequency of Th1, Th17, or Treg cells among these groups (Figures 2A–F). Such data indicated that the *Perp* deficiency did not affect the differentiation of Th1, Th17, or Treg cells in our experimental conditions. Our previous study shows that there is a remarkably inverse correlation between the percentages of IL-17 producing cells and PERP expression in PBMCs from RA patients (16). Meanwhile, the p53 expression was downregulated in PBMC from patients with RA or MS (17). Accordingly, we studied the effect of *Perp^−/−^* on the apoptosis of Th1, Th17, or Treg cells by FACS. While there was no significant difference in the frequency of apoptotic Th1 and Treg cells, the percentages of apoptotic Th17 cells from Lck-Cre × *Perp*^fl/fl^ mice were lower than that from Lck-Cre × *Perp*^fl/+^ and littermate control mice, particularly after anti-Fas treatment (Figures 2G–L). Hence, the PERP may positively regulate Th17 cell apoptosis.

### *Perp^−/−^* Reduces the Caspase Activation in Th17 Cells

**Figure 3:**
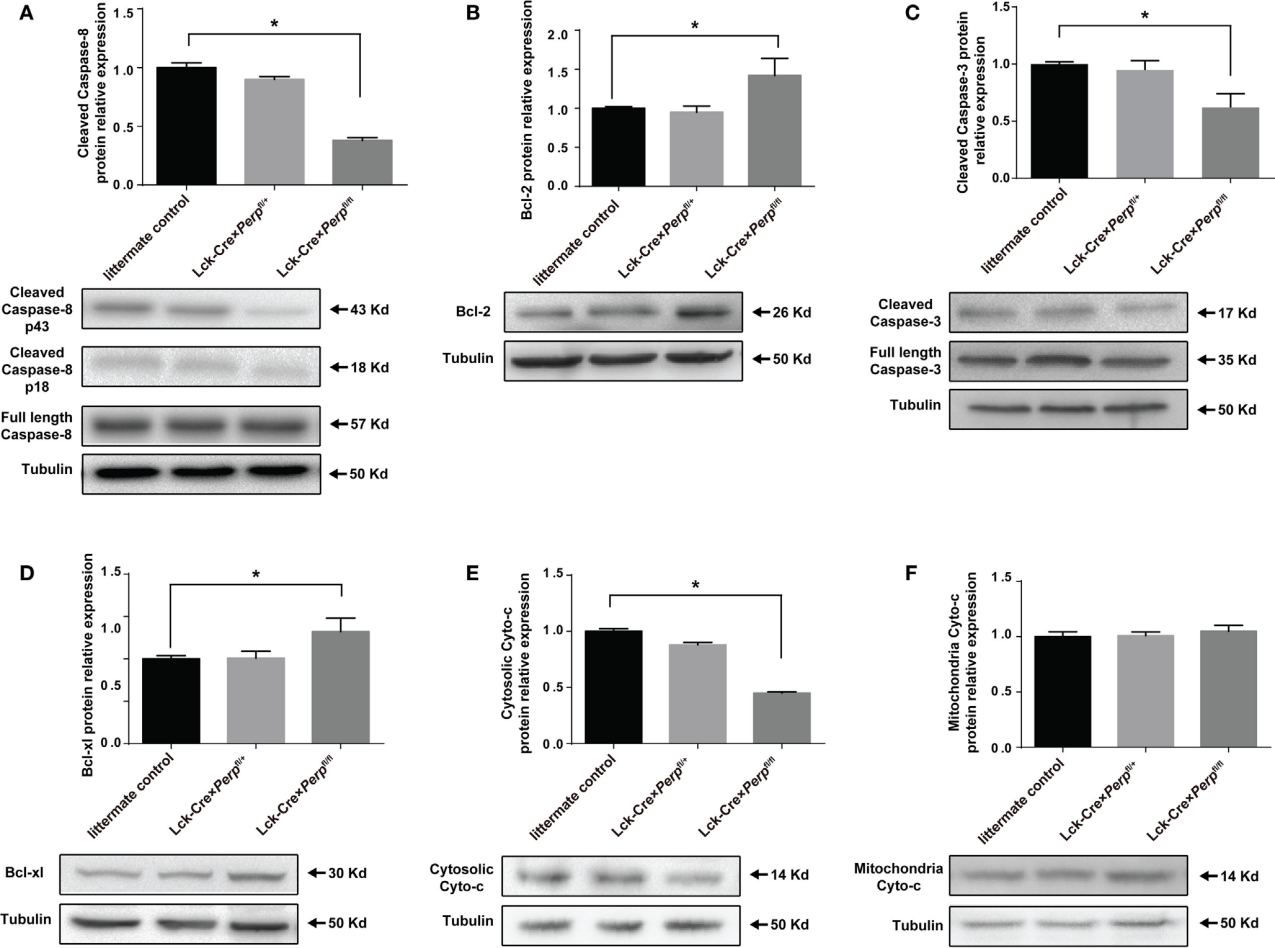
*Perp^−/−^* in T cells promotes the resistance of T helper 17 (Th17) cells to apoptosis in a caspase-dependent manner. Following induction of apoptosis, the relative levels of cleaved caspase-8 **(A)**, Bcl-2 **(B)**, cleaved caspase-3 **(C)**, Bcl-xL **(D)**, cytosolic cytochrome (cyto-*c*) **(E)**, and mitochondrial cyto-*c* **(F)** to the control tubulin expression in the different groups of Th17 cells were determined by Western blot. Data are representative images or expressed as the mean ± SEM of each group from three separate experiments (*P < 0.05).

It is well known that PERP is crucial for caspase activation (18, 19), while Bcl-xL and Bcl-2 are pro-survival factors of Th17 cells (20, 21). To understand the mechanisms underlying the action of *Perp^−/−^* in regulating Th17 cell apoptosis, the relative levels of cyto-*c*, caspase-8, cleaved caspase-3, BcL-x_L_, and BcL-2 expression in the differentiated Th17 cells from three strains of mice were determined by Western blot. There was no significant difference in the relative levels of these molecule expression between heterozygous and littermate control CD4^+^ T cells (Figure 3). However, in comparison with that in the *Perp*^−/+^ and littermate control CD4^+^ T cells, significantly decreased levels of cleaved caspase-8, cytosolic cyto-*c*, cleaved caspase-3, but increased levels of BcL-x_L_ and BcL-2 expression were detected in *Perp^−/−^* CD4^+^ T cells (Figures 3A–E). However, *Perp^−/−^* did not change the levels of mitochondrial cyto-*c* in Th17 cells (Figure 3F). Such data were consistent with previous observation that PERP-induced apoptosis is mediated by the caspase-dependent pathway (18).

### *Perp^−/−^* Enhances the Development of EAE

**Figure 4:**
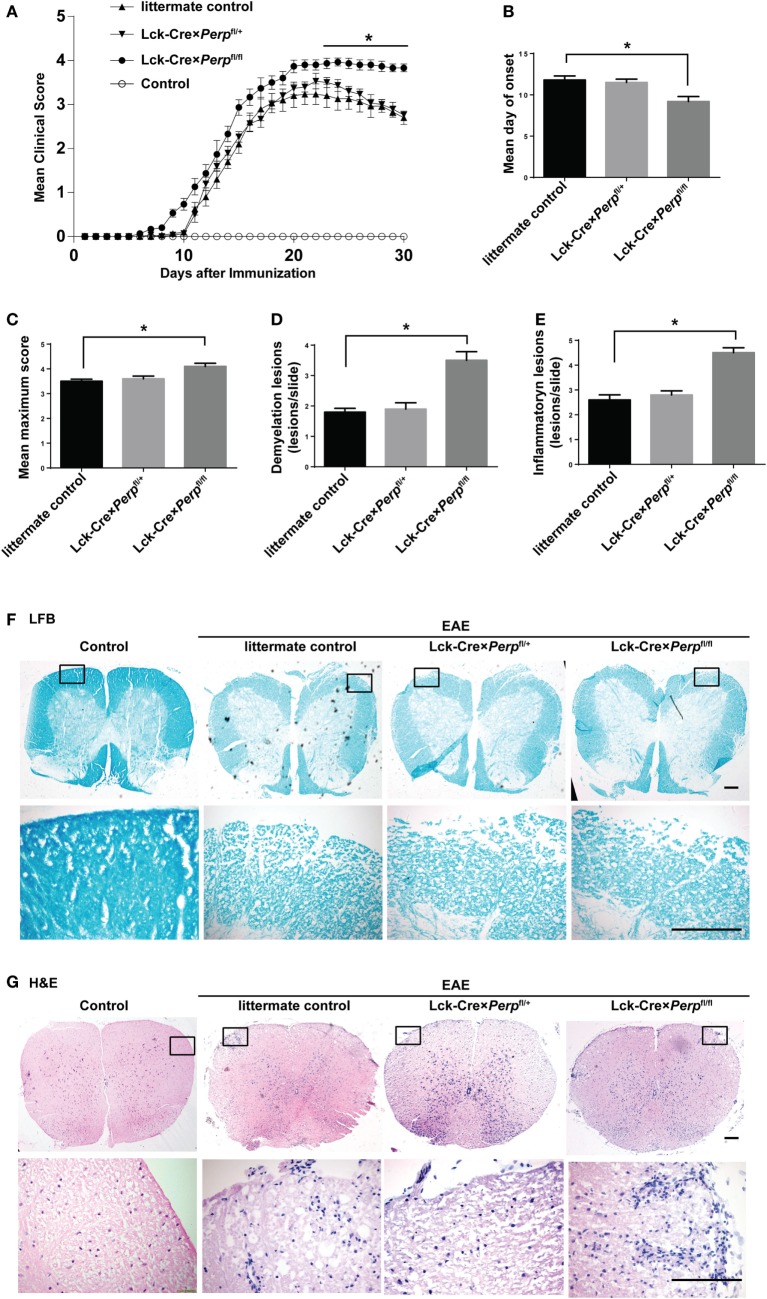
*Perp^−/−^* in T cells exacerbates the development of experimental autoimmune encephalomyelitis (EAE) in mice. Littermate control, Lck-Cre × *Perp*^fl/+^ and Lck-Cre × *Perp*^fl/fl^ mice (*n* = 10 per group) were injected with MOG_35–55_ in 50% complete Freud’s adjuvant and the development and severity of EAE in individual groups of mice were monitored daily **(A)**. The mean time for EAE onset **(B)** and the maximum EAE scores **(C)** in individual groups of mice were calculated. At end of the experiment, the mice were perfused and their spinal cord tissues were dissected, fixed, and stained with LFB **(F)** and H&E **(G)**. The number of lesions with demyelination **(D)** and inflammatory infiltrates **(E)** were quantified in spinal cord section of each mouse. Data are representative images or expressed as the mean ± SEM of each group. Scale bar, 200 µm.

T helper 17 cells play a key role in the development of EAE (22). Given that *Perp^−/−^* decreased Th17 cell apoptosis we investigated that whether *Perp^−/−^* would exacerbate the development of EAE. Littermate control, Lck-Cre × *Perp*^fl/+^ and Lck-Cre × *Perp*^fl/fl^ mice were immunized with MOG_35–55_ in 50% CFA to induce EAE and the severity of EAE in individual groups of mice was monitored daily (Figure 4A). At 23 days post-immunization, the scores of EAE in the Lck-Cre × *Perp*^fl/fl^ mice were significantly higher than that in the littermate control and Lck-Cre × *Perp*^fl/+^ mice. The mean time for disease onset in the Lck-Cre × *Perp*^fl/fl^ mice was significantly earlier than that in the littermate control and Lck-Cre × *Perp*^fl/+^ mice (Figure 4B), and the mean maximum scores the Lck-Cre × *Perp*^fl/fl^ mice were significantly higher than that in the littermate control and Lck-Cre × *Perp*^fl/+^ mice (Figure 4C). Such data indicated that *Perp^−/−^* in T cells exacerbated the development and severity of EAE in mice. Histopathological examinations revealed more obvious demyelination and inflammatory infiltrates in the spinal cord tissues from the Lck-Cre × *Perp*^fl/fl^ mice than that in the littermate control and Lck-Cre × *Perp*^fl/+^ mice (Figures 4D–G).

**Figure 5:**
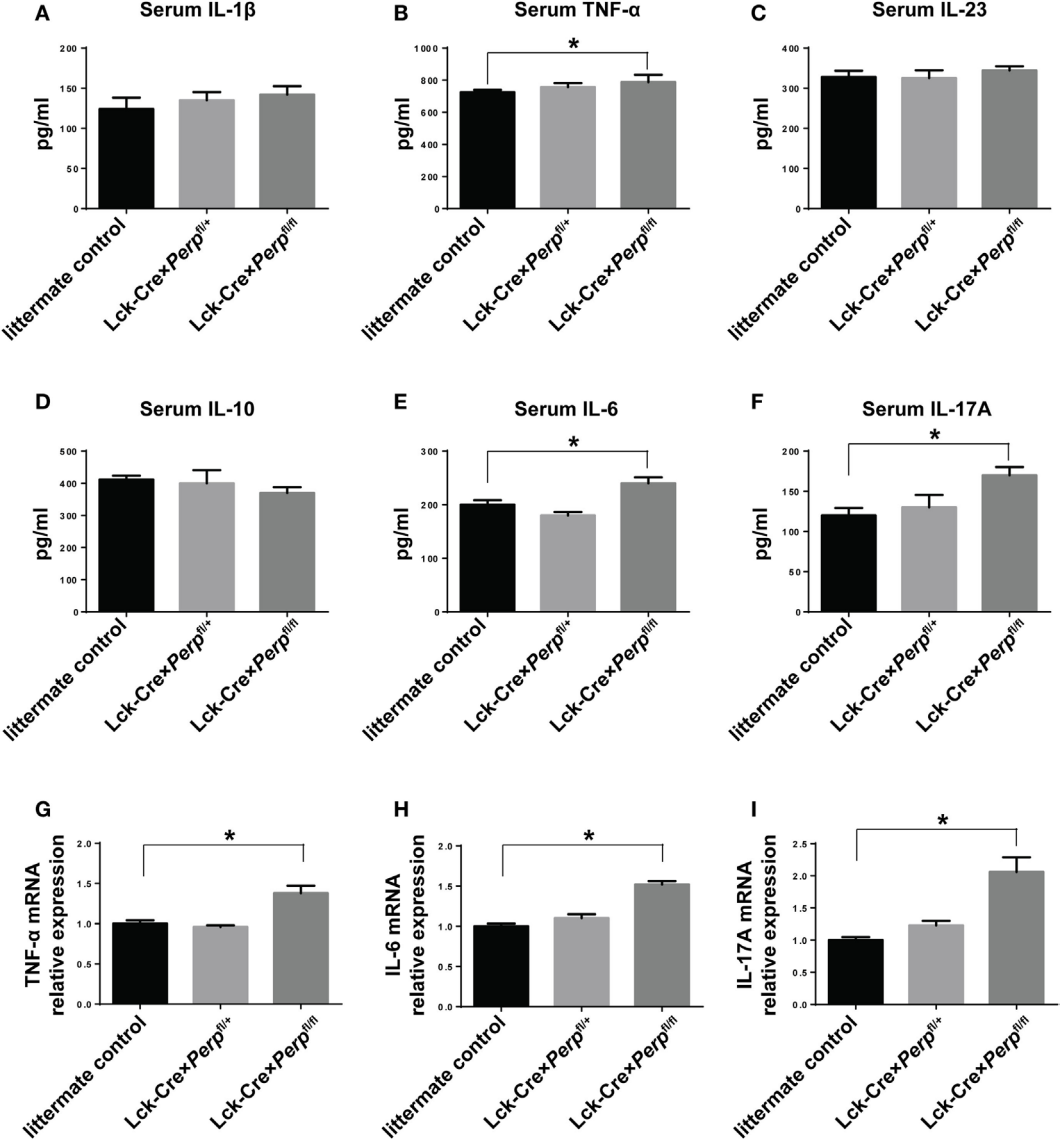
*Perp^−/−^* in T cells increases the levels of pro-inflammatory cytokines in mice following induction of experimental autoimmune encephalomyelitis. At 23 days post-induction, peripheral blood samples and central nervous system tissues were obtained from individual mice. The relative levels of serum IL-1β **(A)**, TNF-α **(B)**, IL-23 **(C)**, IL-10 **(D)**, IL-6 **(E)**, and IL-17A **(F)** were determined by ELISA. The relative levels of TNF-α **(G)**, IL-6 **(H)**, and IL-17A **(I)** mRNA transcripts to GAPDH were determined by quantitative real-time PCR. Data are expressed as the mean ± SEM of each group (*n* = 8 per group) from three separate experiments (*P < 0.05).

**Figure 6:**
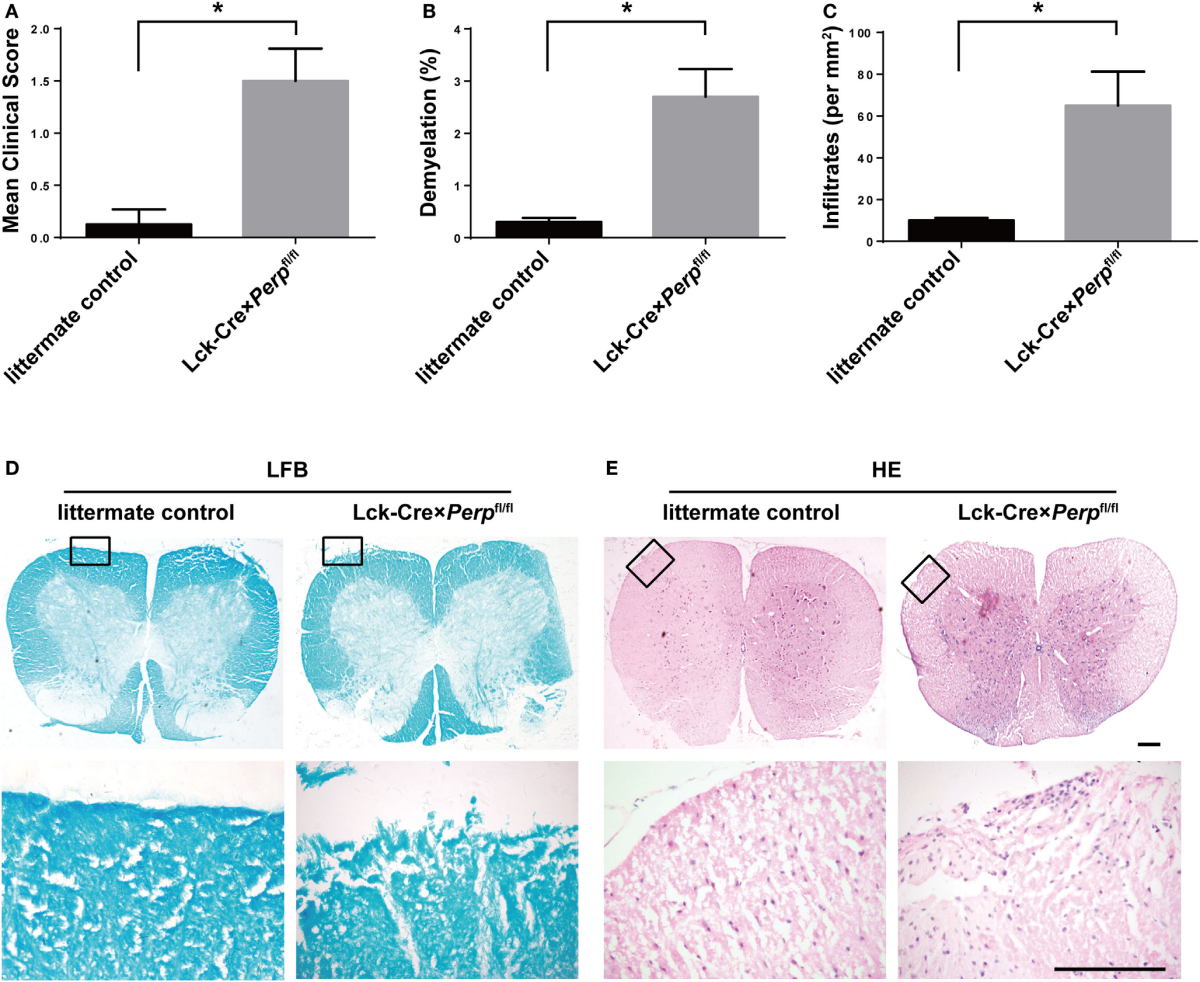
Aged Lck-Cre × *Perp*^fl/fl^ mice exhibit mild experimental autoimmune encephalomyelitis (EAE)-like symptoms. **(A)** The EAE scores of aged Littermate control and Lck-Cre × *Perp*^fl/fl^ mice (*n* = 8 per group, 10 months of age) were calculated. The mice were perfused after symptom onset and their spinal cord tissues were dissected, fixed, and stained with LFB **(D)** and H&E **(E)**. The number of lesions with demyelination **(B)** and infiltrates **(C)** were quantified in three spinal cord tissue sections of each mouse. Data are representative images or expressed as the mean ± SEM of each group. Scale bar, 200 µm.

IL-23, IL-6, and IL-1β are crucial for Th17 cell differentiation and IL-17A and TNF-α are necessary for the development of EAE (23–25). However, IL-10 can inhibit the pathogenicity of Th17 cell-related EAE in mice (26). To further understand the impact of Perp*^−/−^* in T cells on the development of EAE, the levels of serum IL-1β, TNF-α, IL-23, IL-10, IL-6, and IL-17A in individual groups of mice were measured by ELISA. The results showed that the levels of serum TNF-α, IL-6, and IL-17A, but not IL-1β, IL-23, and IL-10, in the Lck-Cre × *Perp*^fl/fl^ mice were significantly higher than that in the littermate control and Lck-Cre × *Perp*^fl/+^ mice (Figures 5A–F). Further quantitative real-time PCR indicated that the relative levels of TNF-α, IL-6, and IL-17A mRNA transcripts to GAPDH in the brain of Lck-Cre × *Perp*^fl/fl^ mice were significantly higher than that in the littermate control and Lck-Cre × *Perp*^fl/+^ mice (Figures 5G–I). In addition, some 10-month-old Lck-Cre × *Perp*^fl/fl^ mice, but not age-matched littermates, developed low degrees of EAE-like symptoms (Figure 6A) and mild demyelination and inflammatory infiltrates in their spinal cord tissues (Figures 6B–E). Together, the early onset, higher disease scores, severer pathogenic lesions, and elevated levels of pro-inflammatory TNF-α, IL-6, and IL-17A demonstrated that *Perp^−/−^* in T cells exacerbated the development and severity of EAE in mice.

### *Perp^−/−^* in T Cells Increases the Frequency of Th17 Cells During the Process of EAE

**Figure 7:**
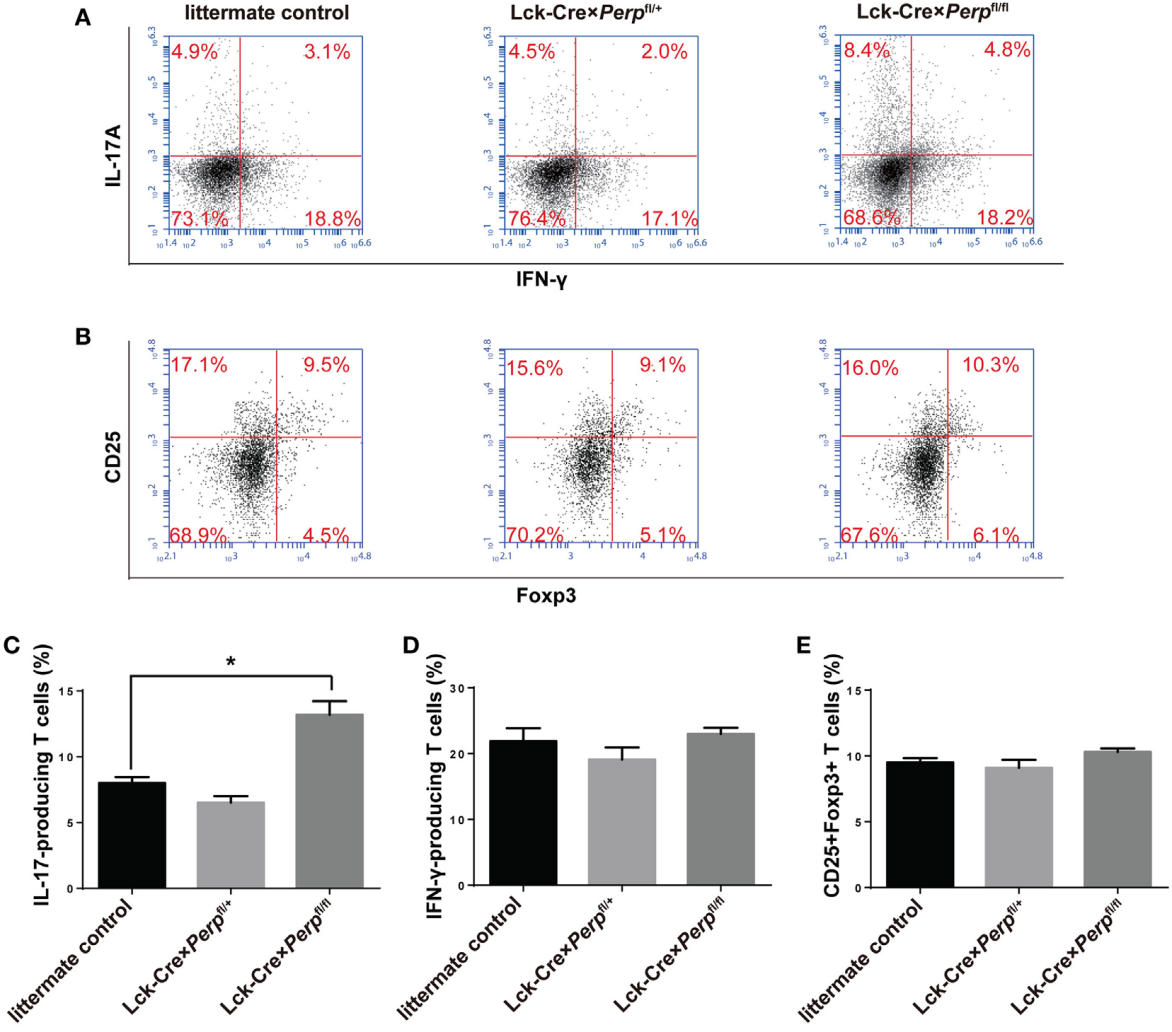

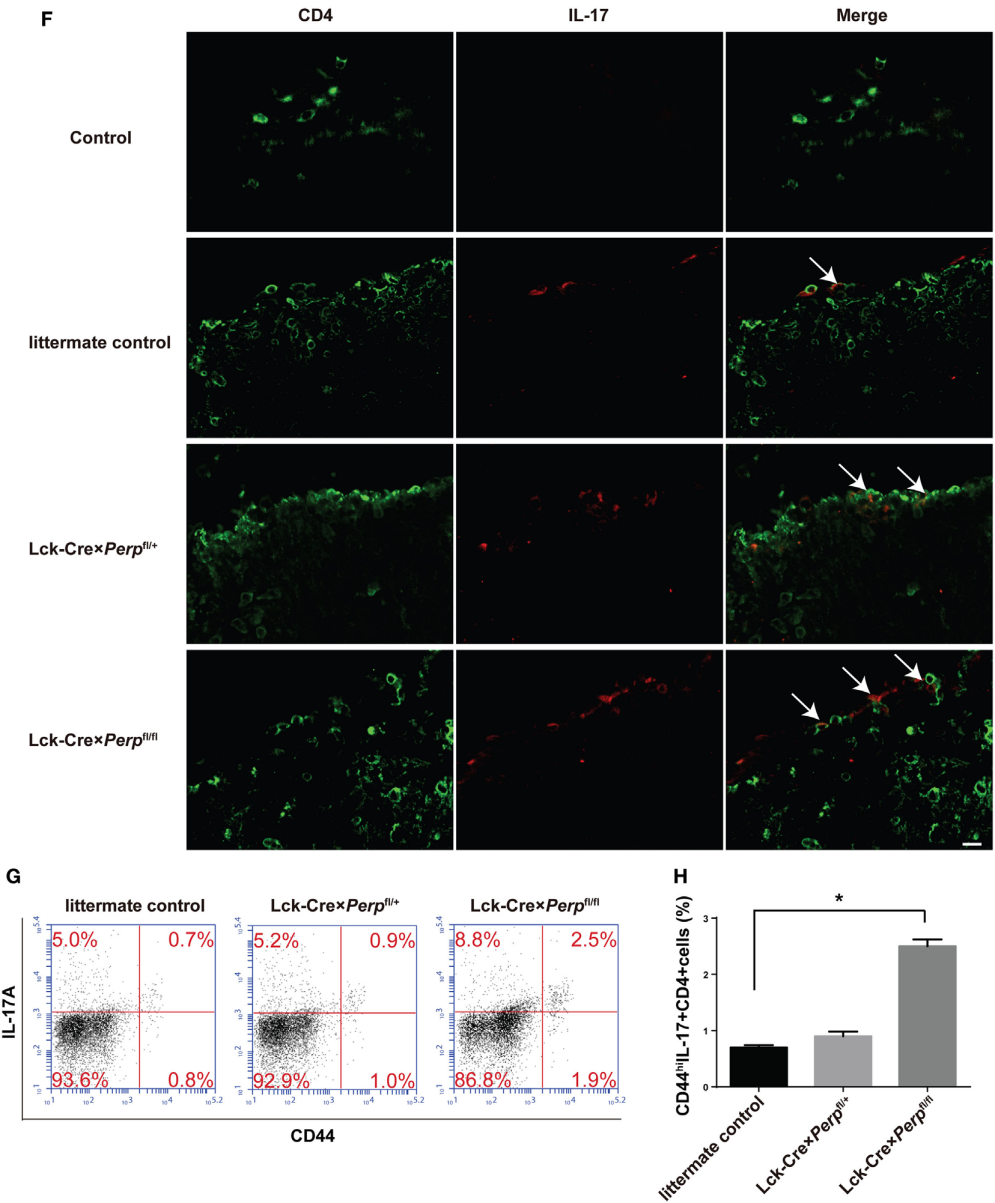
Increased frequency of T helper (Th17) cells in the peripheral spleen and spinal cord tissues in Lck-Cre × *Perp*^fl/fl^ mice. At 23 days post-immunization, splenic mononuclear cells were isolated from littermate control, Lck-Cre × *Perp*^fl/+^ and Lck-Cre × *Perp*^fl/fl^ mice. The cells were stained with anti-CD4, fixed, permeabilized, and intracellularly stained with anti-IFN-γ and anti-IL-17A. Some cells were stained with anti-CD4, anti-CD25, fixed, permeabilized, and intracellularly stained with anti-Foxp3. The percentages of Th1, Th17 cells, and Tregs were determined by FACS. In addition, the presence of Th17 cells in the spinal cord tissues was characterized by immunofluorescent assay. The cryostat spinal cord tissue sections were stained with anti-CD4 (Alexa Fluor 488) and anti-IL-17A (PE), followed by imaging under a confocal microscope. Some splenic cells were stained with anti-CD4, anti-CD44, fixed, permeabilized, and intracellularly stained with anti-IL-17A. The percentages of splenic memory Th17 cells (CD4^+^CD44^hi^IL-17A^+^) were determined by FACS. Data are representative images or expressed as the mean ± SEM of each group (*n* = 8 per group) from three separate experiments. **(A,C,D)** The frequency of Th1 and Th17 cells. **(B,E)** The frequency of Tregs (*P < 0.05). **(F)** Immunofluorescence of CD4 (green) and IL-17 (red) in the spinal cord tissues. Calibration bar, 20 µm. **(G,H)** The frequency of splenic memory Th17 cells.

To understand the mechanisms by which *Perp^−/−^* in T cells exacerbated the development of EAE, the percentages of splenic Th1, Th17 cells, and Tregs in the different groups of mice were characterized by FACS. The results showed that the percentages of Th17 cells, but not Th1 cells and Tregs, in the Lck-Cre × *Perp*^fl/fl^ mice were almost twofold higher than that in the littermate control and Lck-Cre × *Perp*^fl/+^ mice (Figures 7A–E). Further immunofluorescent analysis revealed more frequent IL17A^+^ cells in the spinal cord tissues of the Lck-Cre × *Perp*^fl/fl^ mice than that of the littermate control and Lck-Cre × *Perp*^fl/+^ mice (Figure 7F). Furthermore, the percentages of splenic memory Th17 cells (CD4^+^CD44^hi^IL-17^+^) in the Lck-Cre × *Perp*^fl/fl^ mice were also significantly higher than that in the littermate control and Lck-Cre × *Perp*^fl/+^ mice (Figures 7G,H). Therefore, *Perp^−/−^* in T cells promoted Th17 responses, exacerbating the development of EAE in mice.

## Discussion

Physiologically, PERP functions to regulate the formation of desmosome complex and apoptosis (27, 28). Currently, there is little information on that PERP regulates immune responses because of the postnatal lethality of *Perp^−/−^* mice (27). Our previous study has shown that decreased levels of PERP expression are inversely correlated with disease activity and IL-17A expression in PBMC from patients with RA (16). In this study, we generated the Lck-Cre × *Perp*^fl/fl^ mice with *Perp^−/−^* in T cells and demonstrated that *Perp^−/−^* Th17 cells were relatively resistant to AICD and anti-Fas mediated apoptosis *in vitro* and exacerbated the development of EAE *in vivo* by enhanced Th17 responses. Besides, some aged Lck-Cre × *Perp*^fl/fl^ mice developed mild EAE symptoms. Such data suggest that PERP may promote Th17 cell apoptosis and attenuate the pathogenesis of EAE. Therefore, our findings may provide new insights into the pathogenesis of EAE.

T helper 17 responses are crucial for the pathogenesis of AID, such as MS, systemic lupus erythematosus (SLE), and RA (29, 30). Activated Th17 cells not only exist in peripheral blood but also infiltrate into the inflamed organs during the pathogenic process of SLE (31), rheumatoid synovium (32), and MS (5). It is assumed that the enhanced Th17 responses may be caused by prolonged survival or enhanced differentiation of Th17 cells. In this study, we found that *Perp^−/−^* in T cells did not affect the differentiation of Th1, Th17, or Treg cells. However, *Perp^−/−^* Th17 cells were resistant to apoptosis induced by chronic TCR stimulation or anti-Fas in a caspase-dependent manner. Evidentially, significantly lower levels of cleaved caspase-8 and cleaved caspase-3 were detected in *Perp^−/−^* Th17 cells. Such data support the notion that PERP can activate caspase-8 and caspase-3 to induce apoptosis (18). In addition, we detected significantly higher levels of Bcl-2 and Bcl-xL in *Perp^−/−^* Th17 cells, which should contribute to the survival of *Perp^−/−^* Th17 cells and the exacerbation of EAE (20, 21). It is possible that *Perp^−/−^* may promote the activity of Aiolos and/or c-Myb, which together with the TCR activation-mediated NF-κB activation, enhances Bcl-2 and Bcl-xl expression to support the survival of *Perp^−/−^* Th17 cells (33, 34).

Currently, the survival period of activated Th17 cells remains controversial (35, 36). In this study, we found that *Perp^−/−^* Th17 cells were resistant to apoptosis induced by chronic TCR stimulation and anti-Fas, and increased frequency of peripheral blood Th17 cells was detected in EAE Lck-Cre × *Perp*^fl/fl^ mice. Hence, *Perp^−/−^* supports the survival of Th17 cells although surrounding cells express FasL. These findings may provide a new explanation why decreased levels of PERP expression in PBMC are inversely correlated with Th17 responses in patients with RA (16). More importantly, Lck-Cre × *Perp*^fl/fl^ mice developed severer inflammation and remarkable demyelination in the spinal cord tissues, accompanied by increased levels of pro-inflammatory cytokines and higher frequency of Th17 cells, as well as memory Th17 cells. Given that *Perp^−/−^* in T cells did not affect the differentiation and survival of Th1 cells, it is possible that *Perp^−/−^* in T cells may preferably support the survival of activated Th17 cells, contributing to the development and progression of EAE. It is notable that autoreactive Th17 cells participate in the pathogenesis of several types of AID. Hence, we speculate that *Perp^−/−^* in T cells may also enhance Th17 responses, contributing to the pathogenic process of other AID.

Although *Perp* is a direct transcriptional target of *p53*, previous studies have reported that *p53^−/−^* CD4^+^ T cells differentiate into Th17 more efficiently and *p53* is disposable for inducing the development of Th17 cell-associated autoimmune arthritis (37, 38). Our data showed that *Perp^−/−^* Th17 cells were resistant to AICD and apoptosis induced by anti-Fas. It is notable that *p53^−/−^* mice are prone to development of AID and have strong Th17 responses (39). Actually, a previous study has shown that *p53* negatively regulates AID by inhibiting the Stat3 pathway and suppressing Th17 cell differentiation (40). However, *Perp^−/−^* in T cells did not affect the differentiation of Th17 cells. Apparently, the p53/*Perp* pathway regulates the different stages of the development of Th17 responses in a collaborative manner to control autoreactive Th17 responses and inflammation. Conceptually, enhancement of PERP expression and activity may be valuable for control of autoreactive Th17 responses and AID.

In conclusion, our data indicated that *Perp^−/−^* in T cells did not affect the differentiation of Th1, Th17, or Treg cells, but did promote the resistance of Th17 cells, but not Th1 and Treg cells, to apoptosis induced by chronic TCR stimulation and anti-Fas in a caspase-dependent manner *in vitro*. *Perp^−/−^* in T cells increased the levels of pro-inflammatory cytokines and exacerbated the development of EAE by increased frequency of Th17 cells and severer inflammation and demyelination in the spinal cord tissues of mice. Our findings may highlight a novel function of PERP in the maintenance of Th17 cell homeostasis and new insights in the pathogenesis of EAE. Potentially, enhancement of PERP expression and activity may be a valuable strategy to control AID.

## Ethics Statement

The animal experiment was carried out in accordance with the recommendations of “The Ethics Review Committee for Animal Experimentation at Chengdu Medical College.” The protocol was approved by the “The Ethics Review Committee for Animal Experimentation at Chengdu Medical College.”

## Author Contributions

YZ carried out the histological analysis, the animal experiment and drafted the manuscript. XL and YH carried out the cell culture and FACS. YL (Yan Li) participated in the animal experiment. YL (Yuan Liu) performed Western blotting and ELISA. YL (Yang Liu) and QZ participated in the design of the study and performed the statistical analysis. YW and GS conceived of the study, participated in its design and coordination, and helped to draft the manuscript. All authors read and approved the final manuscript.

## Conflict of Interest Statement

The authors declare that the research was conducted in the absence of any commercial or financial relationships that could be construed as a potential conflict of interest.
